# Supplementary material for: ScITree: Scalable Bayesian inference of transmission tree from epidemiological and genomic data
Source: PLoS Comput Biol. 2025 Jun 10;21(6):e1012657. doi: 10.1371/journal.pcbi.1012657 (PMC12176303; doi:10.1371/journal.pcbi.1012657)
Supplement: S1 Text — 1) Our general MCMC framework for sampling unobserved data and scalar parameters; 2) Additional scenarios for jointly sampling ψj′,Ej′, and δj′ and scenarios in which genetic sampling data is unavailable for a transmission pair; 3) Additional simulations and credible interval coverage rates; 4) Inference of infectious time Ij; 5) Method performance under extreme mutation rates; 6) Performance benchmarking; 7) Computing environment. (PDF) [file pcbi.1012657.s001.pdf]

Supporting Information

S1 Text

General MCMC framework for sampling unobserved data and scalar parameters

We sample our model quantities including infection source, exposure time, and genetic distances for an individual j within a data-augmentation Metropolis-Hastings MCMC framework. The acceptance probability of the jointly-proposed ψ'_j, E'_j, and δ'_j, together denoted Z'_j is

$$p_a = min\{1, \frac{L(Z'_j; y, \theta)}{L(Z_j; y, \theta)} \times \frac{P(Z'_j)}{P(Z_j)} \times \frac{q(Z_j|Z'_j)}{q(Z'_j|Z_j)}\} \quad (1)$$

Where $L(Z'_j; y, \theta)$ is the likelihood of the unobserved data given the observed data and parameters, $\mathrm{P}(Z_j)$ is the prior distribution of Z_j. The equation $q(Z'_j|Z_j)$ is the proposal distribution of Z'_j given the current value of Z_j.

We update ψ'_j, E'_j, and δ'_j sequentially, and the proposal distributions for ψ'_j, E'_j, and δ'_j are assumed to be independent, given the other proposed data values. Thus, $q(Z'_j|Z_j) = q(\delta'_j|\psi'_j, E'_j, \delta_j)q(E'_j|\psi'_j, E_j)q(\psi'_j|\psi_j)$. Likewise, for $q(Z_j|Z'_j)$, $q(Z_j|Z'_j) = q(\delta_j|\psi_j, E_j, \delta'_j)q(E_j|\psi_j, E'_j)q(\psi_j|\psi'_j)$. The parameters in $\theta = (\beta, \kappa, a, b, c, d, \lambda)$ are updated sequentially with a random-walk Metropolis-Hastings algorithm. Using β as an example, a new value β' is proposed from a normal distribution centered at the current value of β with a variance of σ^2_β.

Additional scenarios jointly sampling ψ'_j, E'_j and δ'_j

Our main text describes the key details of our MCMC algorithm using two representative scenarios for joint updating of transmission tree, exposure time, and genetic distances. The specific details of the algorithm are determined by availability of sampled genetic data and the timing of the proposed transmission time. Here, we will give further details of our sampling algorithm under other scenarios and when sampling data is unavailable for a transmission pair.

Our proposal scenarios are broadly divided into two categories based on whether there is sample genomic data available for the source-recipient transmission pair. In the main text, we described sampling for ψ'_j, E'_j and δ'_j for individual j when genomic sampling data is available for the transmission pair and adjacent to the proposed transmission time E'_j. Here, we describe further scenarios which arise, and scenarios in which sampled genomic data is unavailable.

S1 Fig illustrates three scenarios in which genomic data is available for a transmission pair which are not explicitly covered in the main text. We are proposing three genetic distances directly adjacent to the proposed exposure time E'_j: δ_i, δ_k, and δ_j. In scenario A, for source i and infectee j, there is a genomic sample for the source taken at time S_i, before the infectee's proposed exposure time E'_j. However, in this case, there is a transmission event to some other infectee that occurred between S_i and E'_j at time T_i. To propose the genetic distances δ_i, δ_k, and δ_j using the sample data, in a local-greedy algorithm, we subtract any "uninvolved" genetic distances which are not adjacent to E'_j (denoted δ_r in S1 Fig scenario A) to get a "remainder" from the

observed sample distance. Then, we sample δ_i' from that remainder using a binomial distribution, while respecting the current genetic distance δ_i into which we are "inserting" E_j'. The value of δ_i' determines the values of δ_j' and δ_k'. Thus, by this algorithm, we impose the infinite sites assumption locally, in the adjacent genetic distances to E_j'. In S1 Fig scenario B, we show the algorithm for proposing genetic distances δ_i, δ_k, and δ_j when the source genetic sampling time is after E_j'. The algorithm is largely the same as in scenario A. In S1 Fig scenario C, we show the case when genetic sampling data is available and we are inserting a new exposure at time E_j' which is the last event for source i. The proposal is the same as scenario A, however, we only need to propose two genetic distances.

However, the use of the algorithm described in S1 Fig is contingent upon having a genetic sample for each host, between which we can calculate the genetic distance. We now describe our method for sampling the genetic distances when sample data is not available for the source or the infectee in the transmission pair. S2 Fig shows two sampling scenarios when sample genetic distance data is unavailable. In scenario A, we are inserting a proposed exposure at time E_j' in the middle of source i's events. In this case, we propose the genetic distances adjacent to E_j' in the source, δ_i and δ_k, with a binomial draw and we propose the genetic distance in the infectee, δ_j, with a Poisson draw. In S2 Fig scenario B, we are inserting the proposed exposure at the end of source i's events. In this case, we propose the genetic distances adjacent to E_j', δ_i and δ_j, with Poisson draws.

Sensitivity analyses

Additional simulations and credible interval coverage rates

The main manuscript contains results from five representative simulations, here we include additional scalar coverage results and posterior tree coverage rates in S3 Table.

Inference of infectious time I_j

In the main text, we assumed that infectious time for individual j, I_j, is known. Here we investigate the robustness of our method when I_j is not perfectly observed . For the five replicates in the main text, we inferred I_j for each individual, assuming that I_j is known within a two-day time window centered at the true I_j (in practice, this window can be informed by the symptom onset time). We see a small decrease in precision of the estimates, though we still observe a high rate of posterior tree capture, shown in S4 Table. Scalar parameter estimates are displayed in S3 Fig.

Method performance under extreme mutation rates

We simulated data under extreme values of μ_1 and μ_2, where $\mu_1 = 0.004$ and $\mu_2 = 0.001$, for a λ rate of approximately 0.006 base pair mutations per nucleotide base per day (for a sequence of 8,000 base pairs, this is approximately 48 mutations/day). We also simulated data under values of $\mu_1 = 0.01$ and $\mu_2 = 0.01$, for a λ value of approximately 0.03 base pair mutations per nucleotide base per day (for a sequence of 8,000 base pairs, this is approximately 240 mutations/day). Results are shown in S4 Fig and S5 Table. As the mutation rate increases, we see, in general, that the estimation of scalar parameters tends to become less precise, and the mutation rate will be underestimated, suggesting that the infinite-site assumption may be less applicable in these extreme scenarios. However, we see that the transmission tree inference remains relatively robust to a high mutation rate, with source recapture remaining around 90%.

Performance benchmarking

We performed benchmarking for both the genomic and epidemic likelihood portions. We report our benchmarks averaged across 5 simulated datasets with $N = 150$ individuals and $n = 8000$ base pairs in each genetic sequence. We see heavy speed improvement in calculating the genetic likelihood. ScITree, on average, calculates the genomic likelihood in 23.25 microseconds while the Lau 2015 method calculates the genomic likelihood in 3,438.54 microseconds. As expected, we do not see much difference in calculating the epidemiological likelihood: ScITree, on average, calculates the epidemiological likelihood in 75.56 microseconds, while the Lau 2015 method calculates the epidemiological likelihood in 80.35 microseconds. This suggests that the primary factors driving the scalability of this method, as compared to the Lau 2015 method, are the changes made to the genetic likelihood (reflecting the infinite-site assumption).

Computing

We simulate outbreak data (using the Kimura model for the evolutionary dynamics) using the `BORIS` package, which implements the Lau 2015 model in R. [1]. This work is distributed as an R package `ScITree`, developed primarily using `Rcpp` to implement the model inferential algorithm described in our paper [2,3]. An R package can be found at the following link: `https://github.com/hbwddl/ScITree`. Data processing, analysis, and visualizations are done via the `ape`, `coda`, `igraph` and `ggplot2` packages [4–7]. Performance benchmarking was performed with the `RcppClock` package [8].

For each simulated dataset, we ran our inference for a chain of 300,000 iterations, discarding 100,000 as burn-in. For the FMD dataset, we ran three chains of 100,000 iterations, discarding 10,000 as burn-in. We assessed the convergence of scalar parameters through visual inspection of the trace plots and the Geweke diagnostic [9] (all model parameter chains passed at the p=0.01 level). Effective sample sizes of the model parameters are reported in S5 Fig. We see similar effective sample sizes between the Lau 2015 method and ScITree, though the effective sample size for the mutation parameter appears to be higher for ScITree, suggesting that mixing in the Lau 2015 method may be slower due to the need to explore a much larger parameter space.

References

1. Firestone SM, Hayama Y, Lau MS, Yamamoto T, Nishi T, Bradhurst RA, et al. Transmission network reconstruction for foot-and-mouth disease outbreaks incorporating farm-level covariates. PloS one. 2020;15(7):e0235660.

2. R Core Team. R: A Language and Environment for Statistical Computing; 2023. Available from: `https://www.R-project.org/`.

3. Eddelbuettel D, François R. Rcpp: Seamless R and C++ integration. Journal of statistical software. 2011;40:1–18.

4. Paradis E, Schliep K. ape 5.0: an environment for modern phylogenetics and evolutionary analyses in R. Bioinformatics. 2019;35:526–528. doi:10.1093/bioinformatics/bty633.

5. Plummer M, Best N, Cowles K, Vines K. CODA: Convergence Diagnosis and Output Analysis for MCMC. R News. 2006;6(1):7–11.

6. Csardi G, Nepusz T. The igraph software. Complex syst. 2006;1695:1–9.

7. Wickham H. ggplot2: Elegant Graphics for Data Analysis. Springer-Verlag New York; 2016. Available from: https://ggplot2.tidyverse.org.

8. DeBruine Z. RcppClock: Seamless 'Rcpp' Benchmarking; 2021. Available from: https://CRAN.R-project.org/package=RcppClock.

9. Geweke J. In: Bernado JM, Berger JO, Dawid AP, Smith AFM, editors. Evaluating the accuracy of sampling-based approaches to the calculation of posterior moments. Oxford, UK: Clarendon Press; 1992. p. 169–193.
