# Supplementary figures and images for: ScITree: Scalable Bayesian inference of transmission tree from epidemiological and genomic data
Source: PLoS Comput Biol. 2025 Jun 10;21(6):e1012657. doi: 10.1371/journal.pcbi.1012657 (PMC12176303; doi:10.1371/journal.pcbi.1012657)

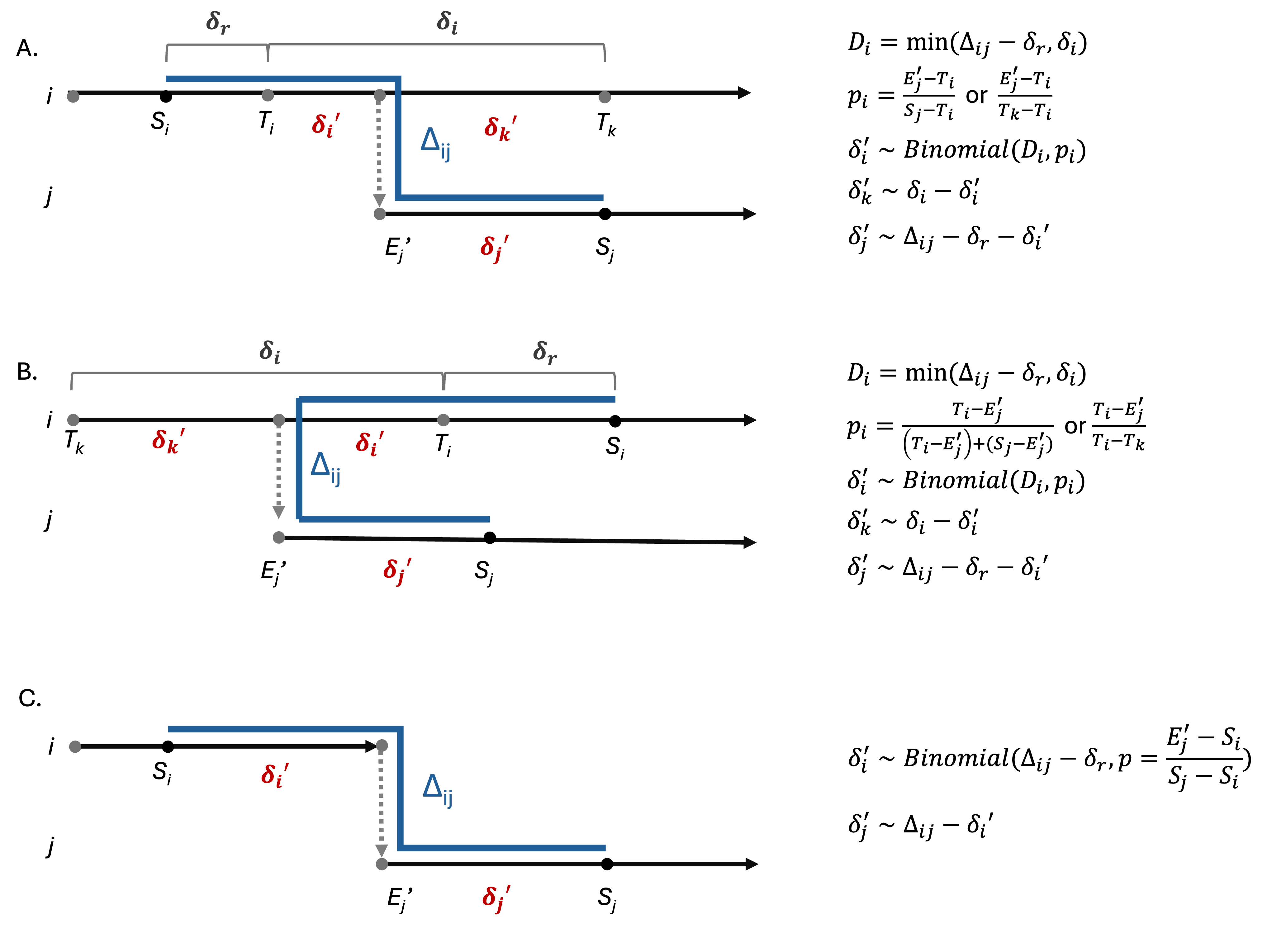

Supplement: S1 Fig — The local greedy algorithm we use respects the infinite-sites assumption for mutations adjacent to the proposed exposure time Ej′. When we have available genomic sampling data, we propose genetic distances adjacent to Ej′, δi,δk, and δj, with a binomial draw from the sample genetic distance, while respecting the current genetic distances in the source i. (TIF) [file pcbi.1012657.s002.tiff]

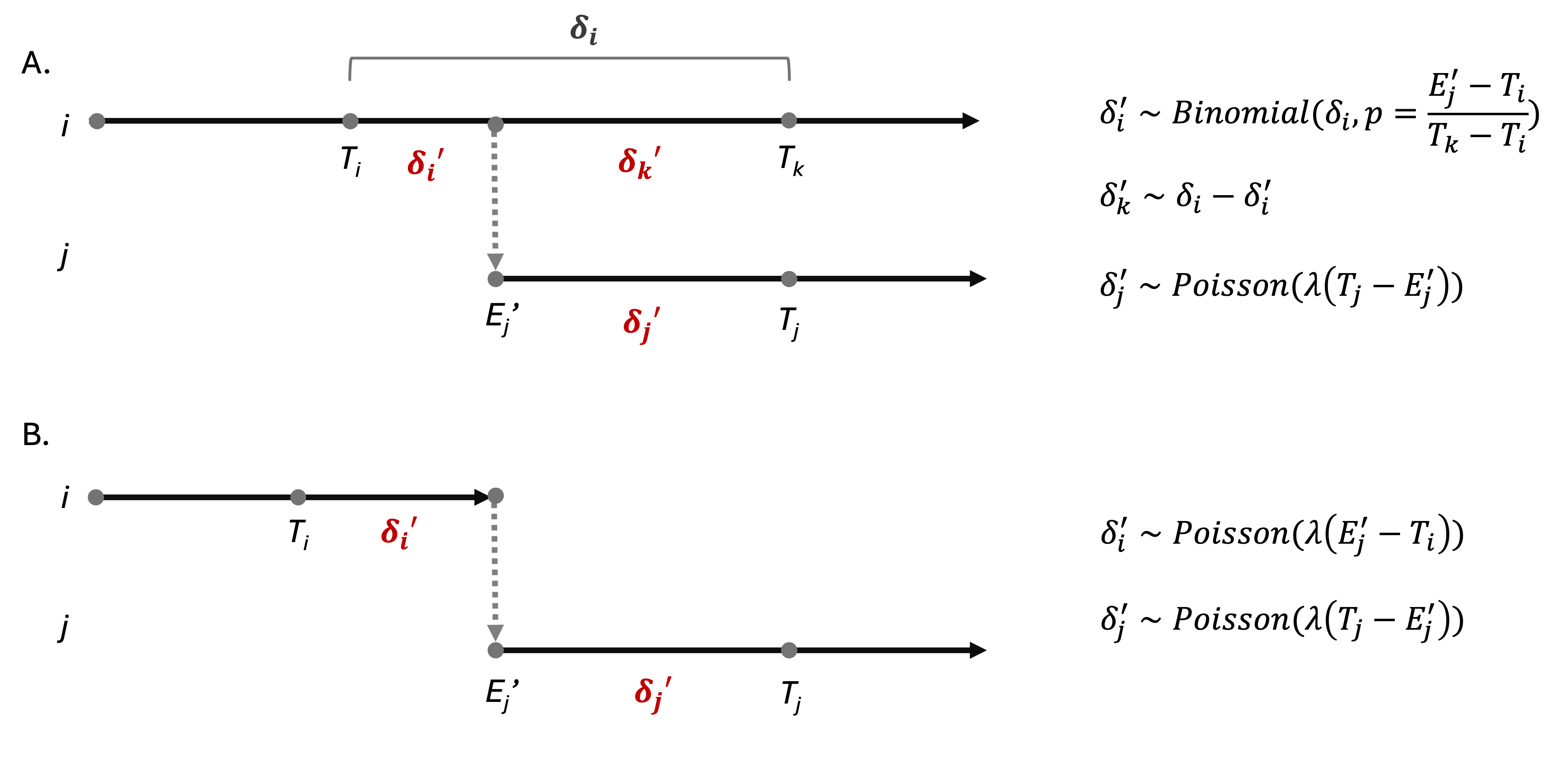

Supplement: S2 Fig — When sample data is not available for the transmission pair i and j, we propose the new genetic distances adjacent to Ej′ with a binomial draw if we are inserting Ej′ into an existing genetic distance (scenario A), or with a Poisson draw using the current value of λ in the MCMC (scenario A, host j, and scenario B). (TIF) [file pcbi.1012657.s003.tiff]

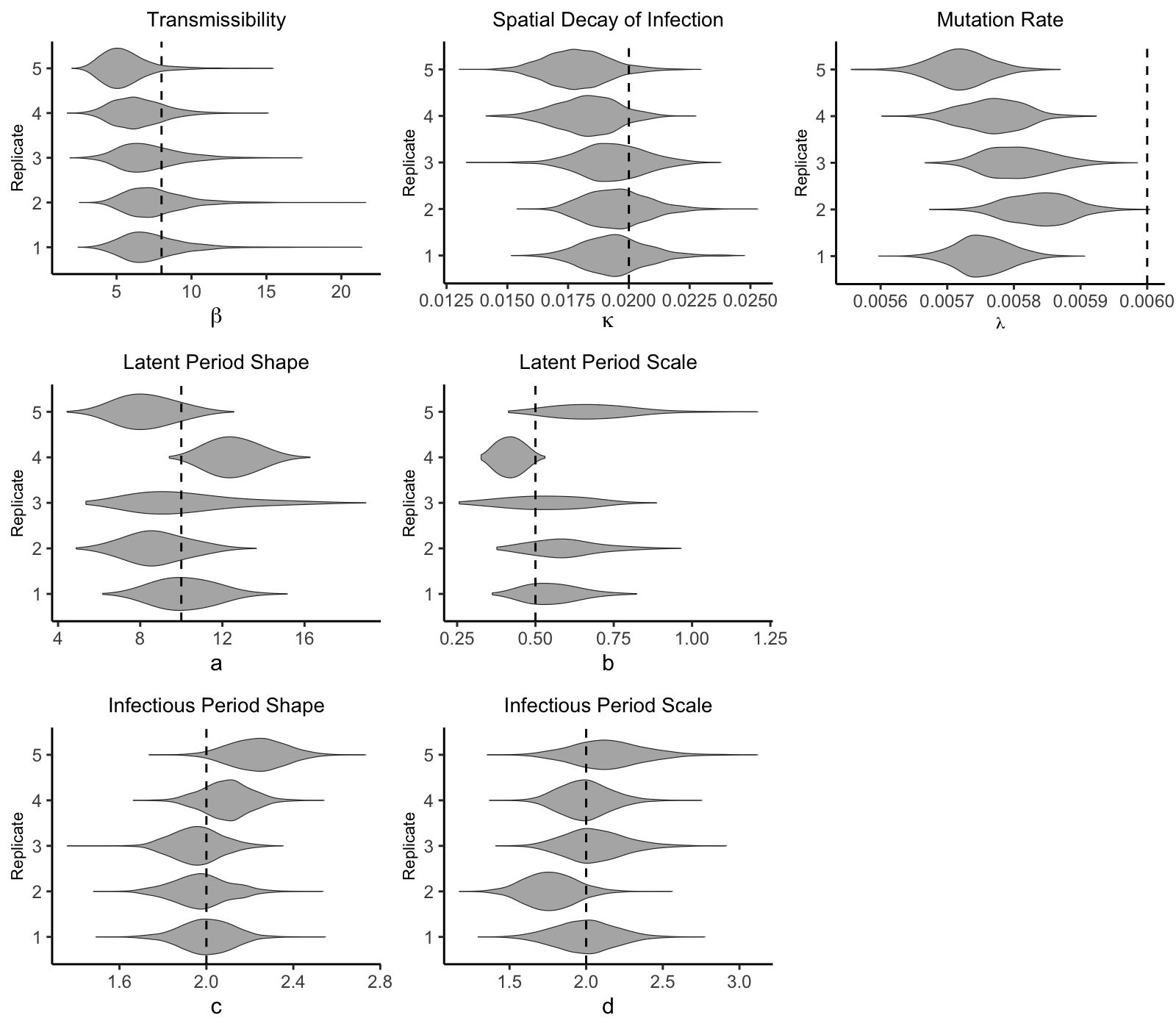

Supplement: S3 Fig — In this case, we assumed that we knew Ij within a 2-day window (which, in practice, may be informed by the symptom onset time). (TIF) [file pcbi.1012657.s004.tiff]

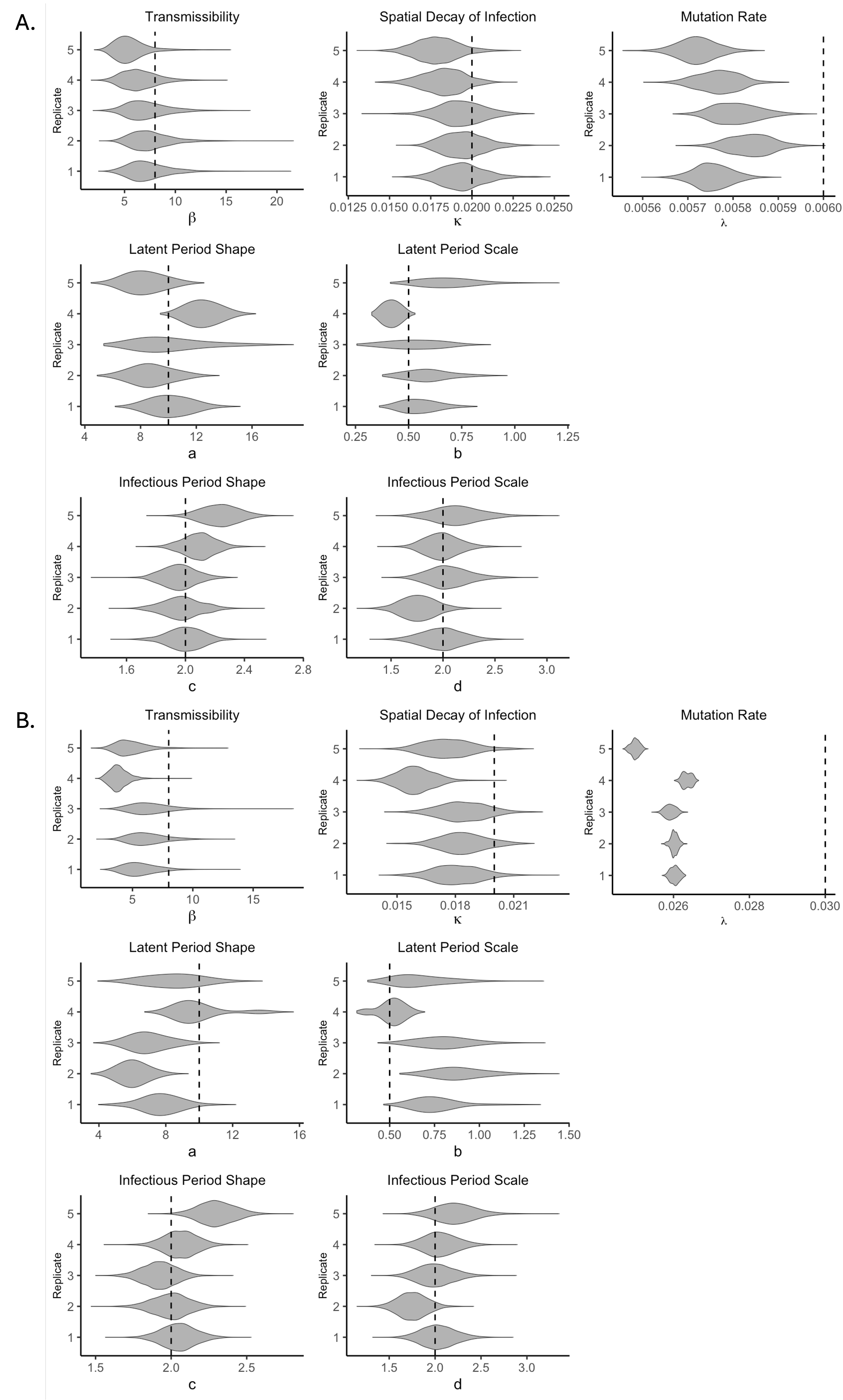

Supplement: S4 Fig — Inference was done for a simulated dataset with 8,000 base pairs in the pathogen genomic data. In figure (a), the mutation rate was λ≈0.006 mutation per base per day, for a mutation rate across the entire sequence of approximately 48 mutations per day. In figure (b), the mutation rate was #x03BB;≈ 0.03 mutations per base per day for a mutation rate across an entire sequence of 240 mutations per day. (TIF) [file pcbi.1012657.s005.tiff]

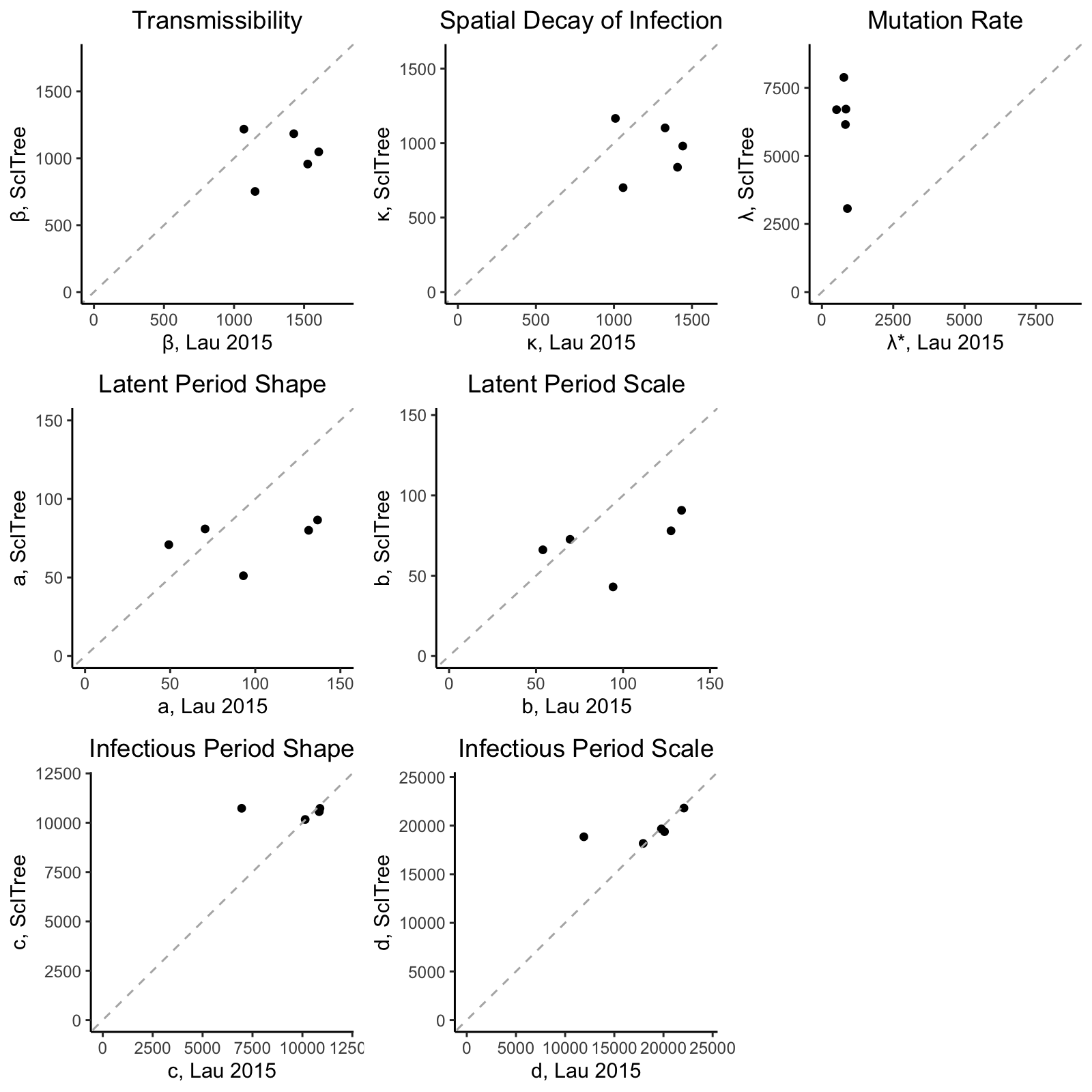

Supplement: S5 Fig — We approximate λ for the Lau 2015 method with the formula λ≈μ1 + 2μ2. (TIF) [file pcbi.1012657.s006.tiff]
