# Supplementary material for: ScITree: Scalable Bayesian inference of transmission tree from epidemiological and genomic data
Source: PLoS Comput Biol. 2025 Jun 10;21(6):e1012657. doi: 10.1371/journal.pcbi.1012657 (PMC12176303; doi:10.1371/journal.pcbi.1012657)
Supplement: S1 Table — (PDF) [file pcbi.1012657.s007.pdf]

Table S1. Prior distributions for model parameters in simulation analyses.

Parameter	Method	Distribution
β (Transmissibility)	ScITree<br>Lau 2015	Unif(0,30)
κ (Spatial kernel)	ScITree<br>Lau 2015	Unif(0,10)
a (Latent period shape)	ScITree<br>Lau 2015	Unif(0,50)
b (Latent period scale)	ScITree<br>Lau 2015	Unif(0.1,50)
c (Infectious period shape)	ScITree<br>Lau 2015	Unif(0,100)
d (Infectious period scale)	ScITree<br>Lau 2015	Unif(0,100)
λ (Mutation rate)(10^{-5})	ScITree	Unif(0,50)
μ_1 (Transition rate)	Lau 2015	Unif(0,10)
μ_2 (Transversion rate)	Lau 2015	Unif(0,1)
