# Supplementary material for: ScITree: Scalable Bayesian inference of transmission tree from epidemiological and genomic data
Source: PLoS Comput Biol. 2025 Jun 10;21(6):e1012657. doi: 10.1371/journal.pcbi.1012657 (PMC12176303; doi:10.1371/journal.pcbi.1012657)
Supplement: S3 Table — (PDF) [file pcbi.1012657.s009.pdf]

Table S3. 95% Credible Interval coverage rate and posterior source coverage rate for 50 simulations. The source coverage rate is the average percent correct source as determined by the most probable posterior source, or the first and second most probable posterior sources.

**Parameter**	**CI Coverage Rate**
β (Transmissibility)	96%
κ (Spatial kernel)	94%
a (Latent period shape)	92%
b (Latent period scale)	90%
c (Infectious period shape)	94%
d (Infectious period scale)	92%
**Source measurement**	**Percent correct (average)**
Most probable source	93.4%
First or second most probable source	98.9%
