# Supplementary material for: ScITree: Scalable Bayesian inference of transmission tree from epidemiological and genomic data
Source: PLoS Comput Biol. 2025 Jun 10;21(6):e1012657. doi: 10.1371/journal.pcbi.1012657 (PMC12176303; doi:10.1371/journal.pcbi.1012657)
Supplement: S4 Table — (PDF) [file pcbi.1012657.s010.pdf]

Table S4. Transmission tree coverage when estimating I_j. The source coverage rate is the percent correct source as determined by the most probable posterior source, or if the correct source is captured in the first or second most probable posterior sources.

**Replicate**	**Correct most probable source**	**Correct first or second most probable source**
1	92.7%	100%
2	93.8%	100%
3	95.6%	99.1%
4	95.4%	100%
5	97.1%	100%
